# Maternal Olfactory Cues Synchronize the Circadian System of Artificially Raised Newborn Rabbits

**DOI:** 10.1371/journal.pone.0074048

**Published:** 2013-09-05

**Authors:** Rodrigo Montúfar-Chaveznava, Lucero Trejo-Muñoz, Oscar Hernández-Campos, Erika Navarrete, Ivette Caldelas

**Affiliations:** 1 Departamento Biología Celular y Fisiología, Instituto de Investigaciones Biomédicas, Universidad Nacional Autónoma de México, Distrito Federal, México; 2 AMITE, Asociación Mexicana para la Innovación en Tecnología Educativa A.C., Distrito Federal, México; Simon Fraser University, Canada

## Abstract

In European newborn rabbits, once-daily nursing acts as a strong non-photic entraining cue for the pre-visual circadian system. Nevertheless, there is a lack of information regarding which of the non-photic cues are capable of modulating pup circadian system. In this study, for the first time, we determined that the mammary pheromone 2-methylbut-2-enal (2MB2) presented in the maternal milk acts as a non-photic entraining cue. We evaluated the effect of once-daily exposure to maternal olfactory cues on the temporal pattern of core body temperature, gross locomotor activity and metabolic variables (liver weight, serum glucose, triacylglycerides, free fatty acids, cholecystokinin and cholesterol levels) in newborn rabbits. Rabbit pups were separated from their mothers from postnatal day 1 (P1) to P8 and were randomly assigned to one of the following conditions: nursed by a lactating doe (NAT); exposed to a 3-min pulse of maternal milk (M-Milk), mammary pheromone (2MB2), or water (H_2_O). To eliminate maternal stimulation, the pups of the last three groups were artificially fed once every 24-h. On P8, the rabbits were sacrificed at different times of the day. In temperature and activity, the NAT, M-Milk and 2MB2 groups exhibited clear diurnal rhythmicity with a conspicuous anticipatory rise hours prior to nursing. In contrast, the H_2_O group exhibited atypical rhythmicity in both parameters, lacking the anticipatory component. At the metabolic level, all of the groups exhibited a diurnal pattern with similar phases in liver weight and metabolites examined. The results obtained in this study suggest that during pre-visual stages of development, the circadian system of newborn rabbits is sensitive to the maternal olfactory cues contained in milk, indicating that these cues function as non-photic entraining signals mainly for the central oscillators regulating the expression of temperature and behavior, whereas in metabolic diurnal rhythmicity, these cues lack an effect, indicating that peripheral oscillators respond to milk administration.

## Introduction

Virtually all organisms exhibit circadian (24-h rhythmic) fluctuations in numerous biological processes, ranging from the behavioral to the molecular level. This temporal organization ensures that vital functions occur in an appropriate sequence and in accordance with cyclic environmental events [1].

The mammalian circadian timing system is organized in a hierarchy of multiple oscillators; the central circadian pacemaker is located in the suprachiasmatic nuclei (SCN) of the anterior hypothalamus and coordinates extra-SCN oscillators, such as the olfactory bulb [2–4], retina [5], lateral habenula [6] and the peripheral oscillators such as the liver, kidney, pancreas, lung and thyroid gland, among others (reviewed in [7]). The functional properties of the pacemaker and oscillators rely to a great extent on a series of feedback loops that drive the cyclical expression of well-known clock genes (reviewed in [8]).

Considerable experimental evidence demonstrate that during early stages of development, the circadian system of altricial mammals is already functional, and it is possible to entrain it to external cyclical cues, mainly to those associated with the mother (reviews in [9,10]). One remarkable example is the European newborn rabbit (*Oryctolagus cuniculus*). This species exhibits 24-h rhythmicity in a range of parameters, at the behavioral [11–13],, physiological [13–16] and molecular levels [16–18]. Maternal care is restricted in rabbits because the mother abandons the nest as soon as she gives birth to the pups and only visits them once every 24 h and nurses for 3 to 5 min [11,19,20]. Thus, the pups remain alone and isolated from environmental signals during the rest of the cycle. This maternal temporal program persists under captivity conditions, providing a remarkable opportunity for the study of the development of circadian rhythmicity without disrupting mother-offspring interaction.

In addition, newborn rabbits anticipate the regular arrival of the lactating female with an increase in general arousal [11–13], body temperature [13,15] and plasma corticosterone levels [21]. This anticipation of nursing persists even if the rabbits are isolated from the mother, indicating that these changes are controlled by the circadian system [13,15]. Interestingly, this anticipatory phenomenon during development has only been reported in rabbits [11–13,15,16,22,23]. It has been widely described in rodents but only during adult life (reviewed in [24,25]).

The once-daily nursing is an effective non-photic entraining signal for the newborn rabbit circadian system because changes in the nursing schedule produce phase shifts in the expression of core body temperature rhythm and in the SCN clock gene expression [16]. During the brief nursing event, pre-visual rabbits are under the influence of complex sensory cues that include tactile, thermal, vestibular, gustatory, ingestive and olfactory information [16]. However, it is currently not clear which of this non-photic cues is capable of acting and modulating the pup circadian system or if the system is sensitive to a combination of these sensory cues.

It is well-known that during pre-visual stages of development, olfaction plays a prominent role for mother-young interaction in mammals [26,27]. In rabbits, the volatile chemical cues originating from the lactating females’ ventrum and milk [27,28] produces a state of arousal in pups, modulates mother-pup interactions and functions as a determinant for nipple location, suckling and orientation to the nest [27–29]. In rabbit milk, a monomolecular signal, the aldehyde 2-methylbut-2-enal (2MB2), also known as mammary pheromone [28] has been identified, which selectively triggers a stereotypical pattern of head searching and oral grasping nipple search behavior in newborn rabbits [28,30]. The responsiveness to 2MB2 does not appear to derive from a prenatal or postnatal learning process, but it is concentration-dependent [31] and species-specific [28].

In the present study, due to the functional significance of the 2MB2 during the early pre-visual stages of development in rabbits, as a first approximation, we determined the role of maternal olfactory cues as non-photic synchronizing signals, investigating the effect of the daily exposure to the 2MB2 in the diurnal expression at the behavioral, physiological and metabolic level in newborn rabbits that were artificially raised.

## Methods

### Study design and animal in-vivo studies

#### Ethics Statement

The experiments were performed according to the National Institutes of Health Guide for the Care and Use of Laboratory Animals (NIH Pub. No. 86-23, revised 1996) and the Treatment of Animals in Research guidelines of the Instituto de Investigaciones Biomédicas, Universidad Nacional Autónoma de México (UNAM). The protocol was reviewed and approved by the Animal Care and Use Committee of the Instituto de Investigaciones Biomédicas, UNAM, prior the conduct of the study (Permit Number: 098).

#### Animals

The Chinchilla strain of domestic rabbits (*Oryctolagus cuniculus*) was used in this study. The animals were bred and maintained at the Instituto de Investigaciones Biomédicas, UNAM. Pregnant rabbits were housed in individual stainless steel cages (120 x 60 x 45 cm) and were maintained on a 16-h light/8-h dark cycle (the lights turned on at 09:00 h). The room temperature was maintained at 20 ± 2 °C with a relative humidity between 40 and 60%; rabbit chow (Conejos Max Premium Reproductor, Malta Cleyton, México) and water were available *ad libitum*.

Four days before the programmed date of parturition, an artificial burrow was placed in the cage that contained the pregnant rabbit. The burrow (28 x 29.5 x 30 cm high) was made of opaque polyvinyl chloride with a 14-cm diameter entrance. Sterile hay was placed in each maternal cage for building nests.

The day of parturition was defined as postnatal day (P) 0. The newborn rabbits were weighed at birth, color-marked on their ears for individual identification and allowed to remain in the burrow with the mother for 6 h. One hundred twenty rabbit pups obtained from 17 litters were used and allocated to one of four groups: pups that had access to a lactating doe (NAT) once every 24-h, olfactory stimulated pups with rabbit maternal milk (M-Milk), olfactory-stimulated pups with the pheromone 2-methyl-but-2enal (2MB2) and olfactory-stimulated pups with water (H_2_O). The last three groups were fed artificially.

To avoid the presence of maternal signals at different moments of the cycle, the rabbit pups were transferred to the recording room isolated from the rest of the colony and placed in cages in groups of four. The cages were made of translucent polysulfide (48 x 27 x 20 cm), which contained paper towel strips and corncob bedding (Argo, México). The rabbits were maintained under constant light conditions (170 lux measured at the top of the cage using a YK-10LX light meter, Lutron, Electronic Enterprise Co, Taiwan); room temperature and relative humidity were maintained at the same ranges previously mentioned throughout the experiment.

The rabbit milk was obtained from lactation does one day after parturition, at 09:00 am. For this purpose the female was immobilized during 5 min and 1.5 ml of maternal milk was manually obtained. Immediately, 150 μl aliquots were made and frozen at -20 ^°^C. For olfactory stimulation, the tube containing the milk was submerged in hot water to 100 ^°^C for 1 min, and the swab was immediately moistened.

#### Olfactory stimulation procedure

To avoid odor contamination, olfactory stimulation was performed in an additional room and in the following order: first, the H_2_O group; next, the M-Milk; and, finally, the 2MB2 pups. From P1 to P8, the pup’s cage was placed inside a hood, and a disposable cotton swab (15.2 cm long) was moistened with 150 μl of distilled water, rabbit maternal milk or 2-Methylbut-2-enal (Tiglic aldehyde, 96%, Sigma-Aldrich, USA) every 24-h (9: 30 am). For olfactory stimulation, the tip of the swab was introduced to the cage approximately 5 cm above the head of the pups for 15 seconds and was removed by 5 seconds and this was repeated for a total of 3 min. After the stimulation, the pups were immediately prepared for feeding, and the hood extraction was maintained for at least 10 min.

#### Feeding procedure

Two feeding strategies were used, namely, normal maternal nursing and orogastric gavage.

For maternal nursing of the NAT group, the newborn rabbits were transferred every 24-h (09:35 am) to the colony room and placed inside the nest box for five min. Immediately after nursing, the pups were removed and transferred to their cages in the recording room.

To eliminate olfactory stimulation and all maternal stimuli that occur during nursing, the pups in the H_2_O, M-Milk and 2MB2 groups were fed by orogastric gavage to deliver the milk formula to the stomach. The milk formula was similar to that previously used by Schley, 1980; Hudson, 1985; Nuesslein-Hildesheim et al., 1995 [32–34]. Milk was delivered by a Silastic tube (0.058 in I.D. and 0.077 in O.D., Dow Corning, USA) coupled to a 20 ml syringe and a 16 G needle. Personnel who were highly trained in the procedure performed the enteral nutrition. From postnatal day 1 to 8, after olfactory stimulation, the pups were gently immobilized with the head and body held vertically for oral gavage, and the milk was slowly perfused to the pup’s stomach. The time required in each pup was approximately 2-4 min, similar to the time that the mother spent to nurse the pups [11]. Immediately, the pups were returned to their cages and to the recording room. The rate of milk formula infusion was increased from approximately 8 ml for 1-day-old pups to approximately 19 ml for 7-day-old pups (approximately 20% of their body weight) according to their body size and stomach filling. The percentage of milk ingested was similar to those previously reported [11,12].

The body weights of all of the animals under study were obtained before and after the feeding procedure. For this reason, previous to the feeding, the urogenital area of the pups were gently rubbed to produce defecation and urination.

### Recordings of gross locomotor activity and core body temperature

The individual rabbits’ locomotor activity and body temperature were recorded simultaneously using telemetry according to previously described methods [13]. On P1, the rabbit pups were anesthetized by the inhalation of Sevoflurane (Sevorane, Abbot, USA) and were implanted i.p. under aseptic conditions with a transponder (G2 E-Mitter, VitalView System, MiniMitter Respironics Inc., USA). After the pups recuperated, they were transferred to the recording room and placed in their cages, and telemetry was performed (ER-4000 Energizer Receiver, MiniMitter Respironics Inc., USA). The data for both parameters were collected in 5-min bins using a *VitalView* telemetry system (Respironics, MiniMitter Inc., USA). The parameters were recorded from P2 to P7.

### Blood and liver sampling and metabolic measures

On postnatal day 8, the pups were sacrificed 1.5 h before the experimental manipulation, and 0, 1.5, 8 and 16 h after the last experimental manipulation (6 animals per time point). To obtain blood and liver samples, the pups were removed from their cages, weighed, placed on a heating pad and anesthetized by the inhalation of Sevoflurane. A thoracotomy was performed to obtain the blood samples. Using a cardiac puncture, a 25 G needle with 1 to 5 ml syringe was inserted into the right ventricle, and 3 ml of blood was collected into test tubes coated with silicone (Kendall, Monojet no. 8881301512). Immediately, the liver was removed and weighed. The blood samples were centrifuged at 3000 rpm for 15 min to obtain blood serum. Aliquots of 1000 μl were coded and frozen at -70°C for subsequent determination of the concentration of glucose (GLU), free fatty acids (FFA), tryacylglycerides (TAG), cholecystokinin (CCK) and cholesterol (CHO). The serum samples were processed by spectrophotometric methods as previously described for rabbit pups [14] using commercial standard enzymatic assay kits (Randox Laboratories LTD, UK and Biosino Bio-technology & Science Inc.). The methods were performed as recommended by the manufacturers.

### Data analysis

The time series obtained for both parameters, that is, core body temperature and gross locomotor activity, were divided into two segments, which were separately analyzed. In the first segment (P2 to P4), in a previous study conducted by our group, we found that the 24-h rhythm was not fully consolidated [13]. In the second segment (P5 to P7), which was considered the pre-visual stage, we found that the 24-h rhythmicity was evident [13].

To evaluate the rhythmicity in the rabbits’ core body temperature and locomotor activity, we used a previously reported procedure by Trejo-Muñoz et al. 2012 [13].

We defined the anticipation as a sustained growth of temperature or activity over time, which crosses or is over a certain threshold. Due to the effect of the developmental increase in temperature (or decrease in activity), we defined the threshold as the 24-h data segment mean. To quantify the anticipatory component, we employed the data corresponding to the first 5 h of each 24-h segment to determine the positions at which the increment began and ended (duration) using the maximum and minimum values as well as the difference between both extreme values (amplitude). Four cases of analysis according to the position of the increment relative to the data mean were presented: (a) it crossed the mean; (b) it was under the mean; (c) it was above the mean; and (d) it was not presented. As mentioned above, only cases (a) and (c) were considered anticipation.

In addition, the mean and standard error were calculated for the daily locomotor activity, core body temperature, phases, and the duration and intensity of the anticipatory component. The differences that were associated with the olfactory treatment (Group) or postnatal day (Age) were tested using two-way ANOVAs for repeated measures followed by the Scheffe post hoc test (significance level 5%).

For the liver weight and metabolic parameters, the data of the four groups under study were separately analyzed using one-way ANOVA for independent measures to determine the potential differences associated with time followed by a COSINOR analysis to evaluate the 24-h rhythmicity, similar to previous studies [16–18]. In addition, the values of the four groups were compared using two-way ANOVAs for the factors of Group and Time followed by the Scheffe post hoc test (significance level 5%).

## Results

### Body weight

The 2-way ANOVA revealed a significant effect of age and among groups on the body weight of the newborn rabbits of the groups under study (Group: F_3, 1181_ = 41.9; *p* = < 0.0001; Age: F_8, 1181_ = 150.6; *p* = < 0.0001; Interaction: F_324, 1181_ = 10.2; *p* = < 0.0001). The initial body weight of the NAT group was 52.6 ± 8 g at P0, reaching a weight of 116.2 ± 21.9 g at the end of the experiment. In contrast, the pups that were fed artificially and olfactory stimulated with M-Milk, 2MB2 and H_2_O groups exhibited an initial weight of 54.1 ± 8.5, 55.5 ± 9 and 53.9 ± 11.2 g, respectively, at P0. At P8, the animals reached a weight of 82.9 ± 12.4, 83.5 ± 14.5 and 80.9 ± 17.6 g, respectively. At the beginning of the experiment, all of the animals exhibited similar body weights. However, at the end of the experiment, a significant lack of gain in the body weight of the newborn rabbits fed by enteral nutrition by approximately 28-30% in relationship to the pups normally nursed was evident.

### Core body temperature

**Figure 1:**
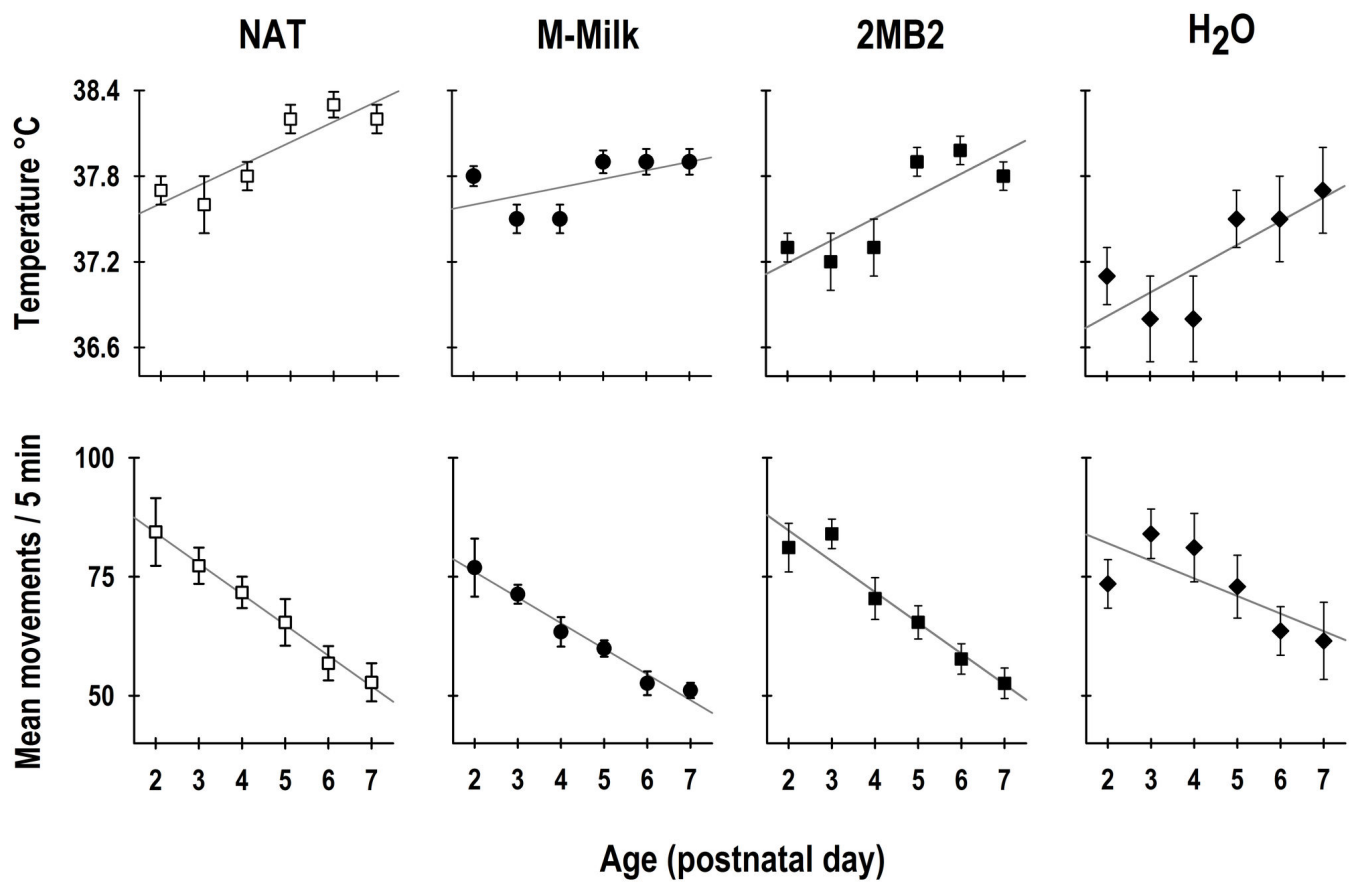
Daily average of core body temperature and gross locomotor activity. The daily core body temperature (top panel) and gross locomotor activity (bottom panel) of newborn rabbits that were maintained under constant light and received one of the following treatments: once every 24-h had access to a lactating doe (NAT), or fed artificially and were olfactory stimulated with either rabbit maternal milk (M-Milk), pheromone 2-methyl-but-2enal (2MB2) or with water (H_2_O) from postnatal day 2-7. Mean ± SEM. For body temperature: NAT, r^2^ = 0.79; M-Milk, r^2^ = 0.32; 2MB2, r^2^ = 0.69; H_2_O, r^2^ = 0.63. For locomotor activity: NAT, r^2^ = 0.99; M-Milk, r^2^ = 0.97; 2MB2, r^2^ = 0.94; H_2_O, r^2^ = 0.56.

A 2-way ANOVA revealed significant effects associated with the experimental manipulation and age in the average temperature of newborn rabbits (Group: F_3,164_ = 12.9; *p* = < 0.0001; Age: F_5,164_= 7.3; *p* = < 0.001; Interaction: F_15,164_= 0.3; *p* = NS). The average daily core body temperature of the rabbit pups exhibited a significant increase consistent with age (Figure 1, top panel). The mean temperature of the NAT pups on P2 was 37.7 ± 0.1 ^°^C, which increased gradually to reach 38.2 ± 0.1 ^°^C on P7. Similarly, the newborn rabbits that were exposed to the maternal olfactory cues, the pups of the M-Milk and 2MB2 groups, exhibited an initial core temperature 37.8 ± 0.08 ^°^C and 37.4 ± 0.1 ^°^C, respectively. At the end of the experiment on P7, the temperature was 37.9 ± 0.09 ^°^C and 37.8 ± 0.1 ^°^C, respectively. In contrast, the pups of the H_2_O group showed an initial core temperature of 37.1 ± 0.2 ^°^C, reaching a final temperature of 37.6 ± 0.3 ^°^C on P7. The pups of the H_2_O group showed a significant decrease of 0.6 ^°^C in the daily average of the core body temperature compared to the NAT group but also in relationship to the other enteral feeding groups that received the olfactory stimulation (Figure 1, top panel).

**Figure 2:**
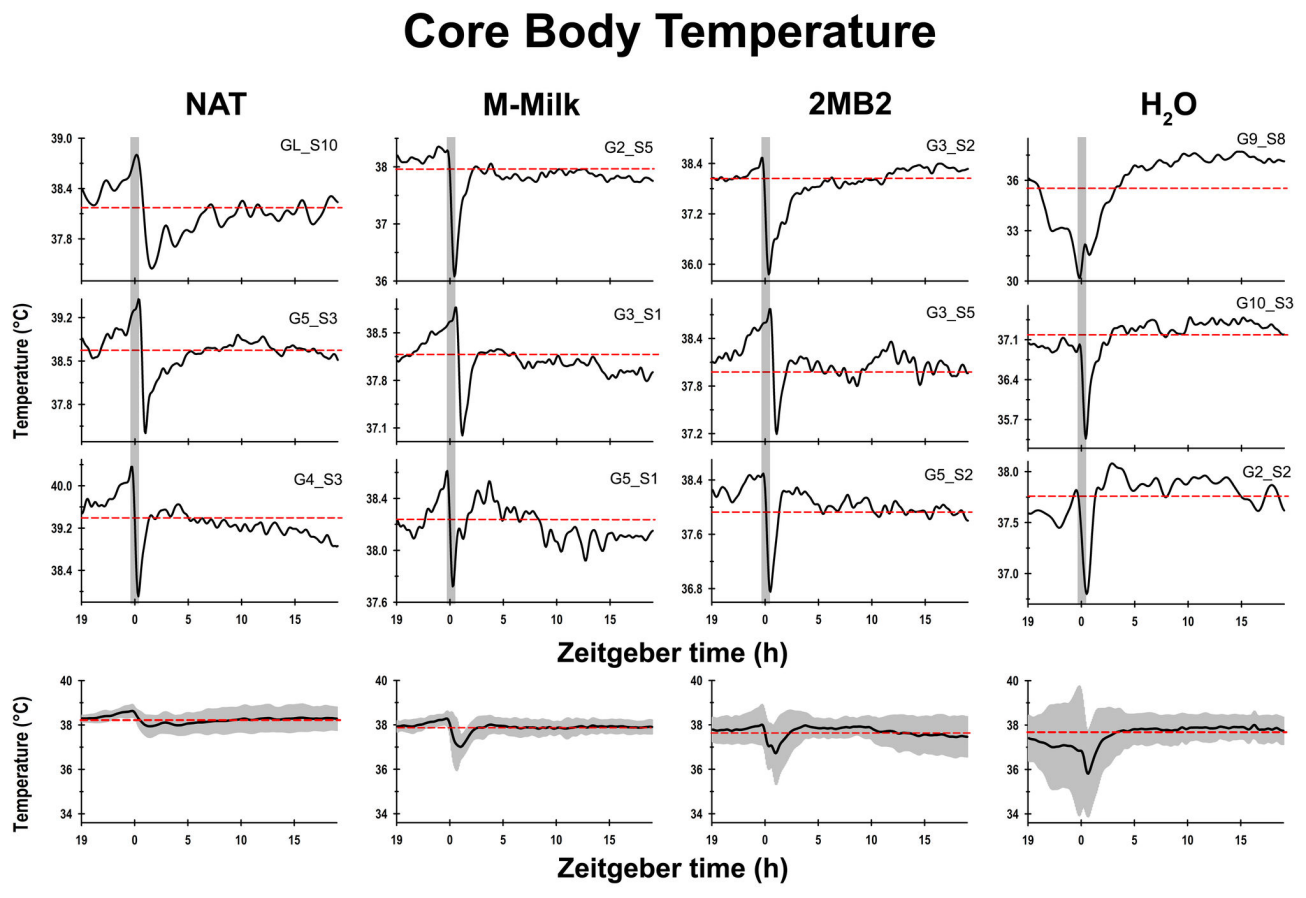
Diurnal rhythm of core body temperature. Representative temporal profiles of the core body temperature as measured using biotelemetry of the newborn rabbits on postnatal day 7, which were maintained under constant light conditions and received the following treatment: pups that once every 24-h (indicated by the vertical gray bar) had access to a lactating doe (NAT), or olfactory stimulated with rabbit maternal milk (M-Milk) or with the pheromone 2-methyl-but-2enal (2MB2) or with water (H_2_O). At the bottom, the mean diurnal pattern of body temperature and standard error (SEM) of each experimental condition from postnatal days 5 to 7 for all pups (eight animals per group) examined.

With respect to the temporal pattern of the core body temperature, the NAT rabbits exhibited a characteristic diurnal rhythm (Figure 2), in which its core body temperature started to rise above the 24-h mean hours before the scheduled time of access to the lactating doe for nursing, and following this episode, their average body temperature dropped almost immediately below the 24-h mean and remained at this low level for approximately 5 h. The M-Milk and 2MB2 groups exhibited close similarities of the temporal profile of the core temperature to the one observed in the NAT group. Specifically, these pups exhibited a rise above the 24-h mean h before the scheduled time of maternal odor exposition followed by a marked drop in temperature for approximately 3 to 5 h (Figure 2). All the newborn rabbits of the H_2_O group exhibited an atypical temporal pattern of core body temperature, which considerably differed from the rest of the groups examined. Five pups of the H_2_O group exhibited temperatures below the 24-h temperature mean approximately 5 h before the scheduled time of olfactory H_2_O exposition. Even during the experimental manipulation, the core body temperature remained below the 24-h mean. The remaining animals (n=3), showed a modest increase to the 24-h mean temperature minutes prior to the olfactory stimulation with H_2_O. After the manipulation, all of the animals exhibited a marked drop in temperature for approximately 1 to 3 h (Figure 2).

**Figure 3:**
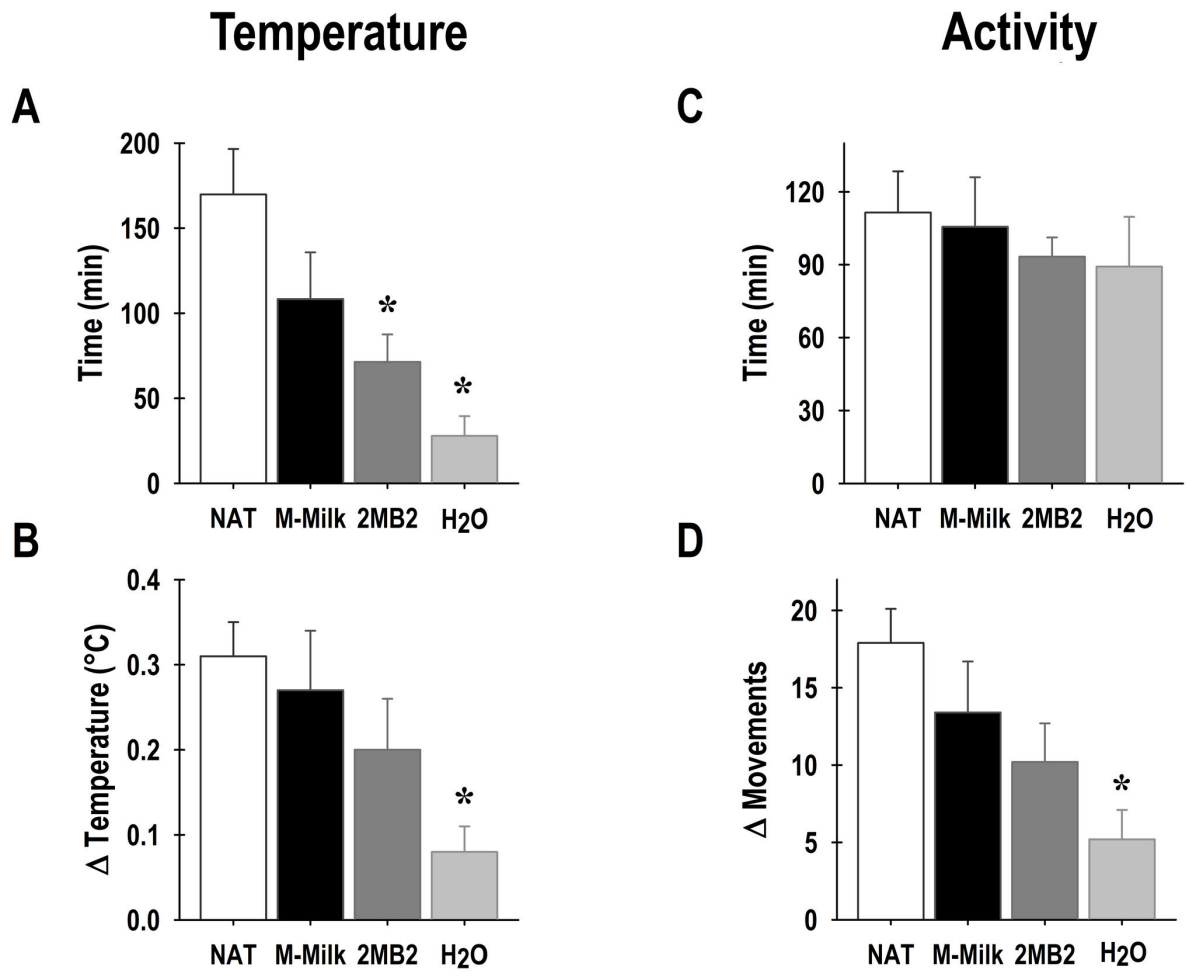
Duration and magnitude of the anticipatory component, in body temperature and locomotor activity. Graphics of the duration of the anticipation in core body temperature (A) and gross locomotor activity (C) of the newborn rabbits on postnatal day 7. Pups were maintained under constant light and received one of the following treatments: once every 24-h had access to a lactating doe (NAT), or olfactory stimulated with rabbit maternal milk (M-Milk), or with the pheromone 2-methyl-but-2enal (2MB2) or with water (H_2_O). Graphics of the intensity of the anticipatory increase in relationship to the daily mean core temperature (B) and locomotor activity (D) for each group examined. Mean ± SEM. Scheffe * *p*<0.01 vs. NAT.

Significant changes associated with olfactory stimulation were observed in the duration of the pups’ anticipation of nursing (F_3,26_ = 7.1; p<0.001). On P7, the NAT pups exhibited an anticipatory rise in temperature of 168 ± 24 min, similar to the M-Milk pups, which showed an anticipatory component of 108 ± 30 min (Figure 3, panel A). The pups that were stimulated with the mammary pheromone 2MB2 and those stimulated with H_2_O showed a significant decrease in the anticipatory rise in temperature of 72 ± 18 min and 24 ± 12 min, respectively, compared to the NAT group. The decrease in the duration of the anticipatory component was more conspicuous in the H_2_O rabbit pups (Figure 3, panel A). Similarly, significant changes associated with olfactory stimulation were detected for the intensity of the anticipatory rise in core body temperature (F_3,26_ = 3.3; p<0.03). On P7, similar temperature increases were observed during the anticipatory rise in the NAT, M-Milk and 2MB2 groups, which were 0.31 ± 0.03 ^°^C, 0.27 ± 0.07 ^°^C and 0.2 ± 0.06 ^°^C, respectively, in relationship to the average temperature (Figure 3, panel B). In contrast, the H_2_O rabbit pups exhibited a significant decrease in the magnitude of the anticipatory rise 0.08 ± 0.03 ^°^C compared to the NAT rabbit pups (Figure 3, panel B).

### Locomotor activity

A 2-way ANOVA revealed significant effects associated with the experimental manipulation and age in the daily average of gross locomotor activity of newborn rabbits (Group: F_3,164_ = 4.8; *p* = < 0.003; Age: F_5,164_= 19.9; *p* < 0.0001; Interaction: F_15,164_= 0.6; *p* = NS), which exhibited a significant decrease that was consistent with age (Figure 1, bottom panel). The mean activity of the NAT pups on P2 was 84.4 ± 7.1 movements, which decreased gradually to 52.8 ± 4.0 movements on P7. Likewise, the newborn rabbits that were exposed to the maternal olfactory cues, the pups of the M-Milk and 2MB2 groups, exhibited an initial activity of 76.9 ± 6.2 and 81.1 ± 5.2 movements, respectively. At the end of the experiment on P7, the average activity was 51.1 ± 1.7 and 52.6 ± 3.3 movements, respectively. In contrast, the pups of the H_2_O group showed an initial locomotor activity of 73.5 ± 5.2 movements, which decreased on P7 to 61.5 ± 8.1 movements (Figure 1, bottom panel).

**Figure 4:**
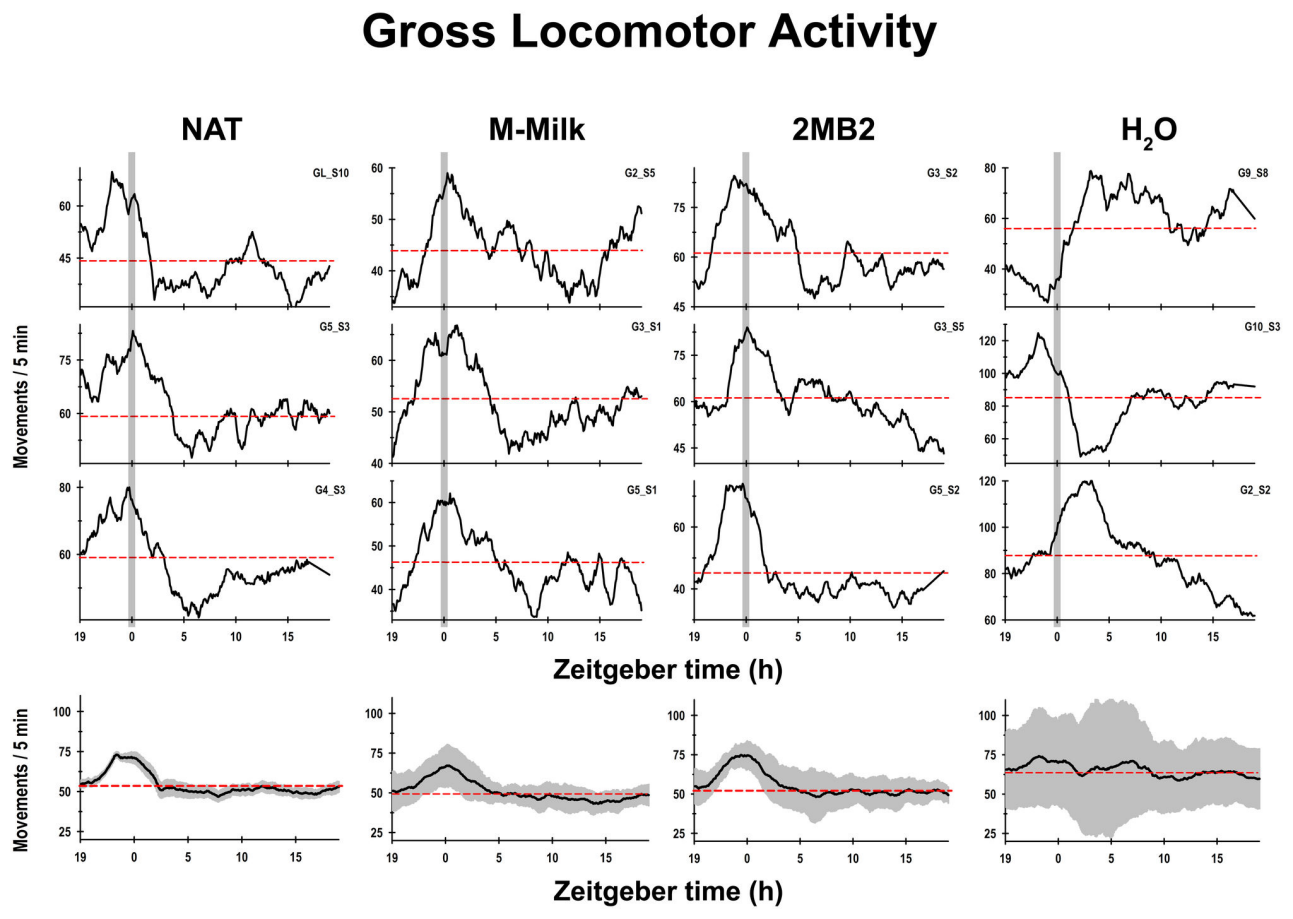
Diurnal rhythm of gross locomotor activity. Representative temporal profiles of gross locomotor activity as measured using biotelemetry of newborn rabbits on postnatal day 7, maintained under constant light conditions and receiving the following treatment: pups that once every 24-h (indicated by the vertical grey bar) had access to a lactating doe (NAT), or olfactory stimulated with rabbit maternal milk (M-Milk) or with the pheromone 2-methyl-but-2enal (2MB2) or with water (H_2_O). At the bottom, the mean diurnal pattern of body temperature and standard error (SEM), of each experimental condition from postnatal days 5 to 7 for all of the pups (eight animals per group) examined.

The NAT pups exhibited a characteristic diurnal rhythm in which the gross locomotor activity started to rise above the 24-h mean hours before the scheduled time of access to a lactating doe, and following this episode, the activity decreased gradually below the 24-h mean, and remained at this low level for approximately 6-8 h (Figure 4). The M-Milk and 2MB2 pups exhibited close similarities in the activity temporal profile to the one observed in the NAT group with a rise in activity above the 24-h mean hours before the scheduled time of maternal odor exposition followed by a marked decrease in activity for approximately 6-8 h (Figure 4). Interestingly, all of the pups of the H_2_O group exhibited an atypical temporal pattern of gross locomotor activity that differed considerably from what was observed in other groups under study. In some cases, the H_2_O pups exhibited bimodal patterns in their locomotor activity. In addition, five of the eight pups of this group presented an activity level below the 24-h mean approximately 5 h before the scheduled time of olfactory H_2_O stimulation. After the experimental manipulation, six animals showed a significant increase above the 24-h mean in activity for approximately 7 to 10 h, and two animals exhibited a marked decrease in activity after the olfactory H_2_O exposition for approximately 7 h (Figure 4). In addition, a remarkable increase in the variation in the mean diurnal pattern of locomotor activity among the H_2_O pups was observed (Figure 4).

At the behavioral level, no significant effects were observed in the duration of the anticipation to nursing (F_3,26_ = 0.3; p= NS) in the groups examined (Figure 3, panel C). On P7, the NAT pups showed an anticipatory increase in locomotor activity for 114 ± 18 min. A similar tendency was observed in the M-Milk, 2MB2 and H_2_O pups, in which the anticipatory component lasted for 108 ± 18 min, 96 ± 6 min and 90 ± 18 min, respectively (Figure 3, panel C). In contrast, significant changes were detected regarding the intensity of the anticipatory increase in the locomotor activity of rabbit pups (F_3,26_ = 4.2; p<0.01). On P7, the anticipatory activity increase (in relation to the average activity) in the NAT, M-Milk and 2MB2 groups, were 17.9 ± 2.1, 13.4 ± 3.3 and 10.2 ± 2.5 movements, respectively (Figure 3, panel D). Importantly, the H_2_O rabbit pups exhibited a significant decrease in the magnitude of the anticipatory rise at 5.2 ± 1.8 movements compared to the NAT rabbit pups (Figure 3, panel D).

### Liver weight and metabolic parameters

**Figure 5:**
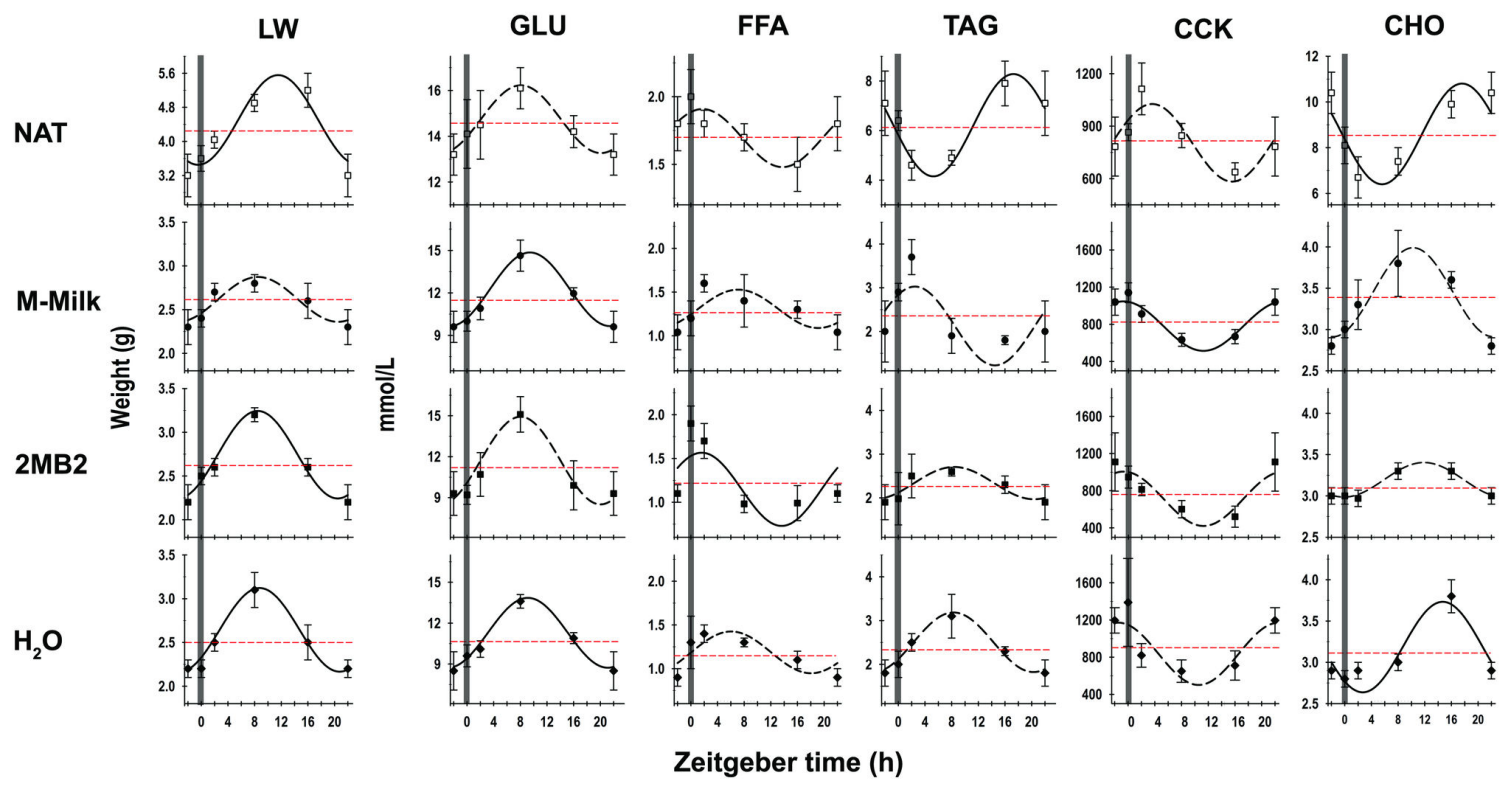
Metabolites temporal pattern. Temporal profile of: liver weight (Liver) and serum levels of glucose (GLU), free fatty acids (FFA), tryacylglycerides (TAG), cholecystokinin (CCK) and cholesterol (CHO) of newborn rabbits on postnatal day 8, that were maintained under constant light, and received one of the following treatments: had access to a lactating doe (NAT) once every 24-h (indicated by the vertical gray bar) or olfactory stimulated with rabbit maternal milk (M-Milk), with the pheromone 2-methyl-but-2enal (2MB2) or with water (H_2_O). Mean ± SEM, and the lines showed the fitted Cosinor function (see text for details). Mesor is indicated by the horizontal dotted line.

The one-way ANOVA and COSINOR analysis (Tables 1 and 2, respectively) revealed that the liver exhibited significant diurnal weight fluctuations in all groups of rabbit pups examined with an exception of the M-Milk group, which only showed a tendency of 79.7% for rhythmicity (Figure 5). All of the groups examined exhibited close similarities in the 24-h temporal pattern in liver weight, with the minimum values occurring 2 h before the experimental manipulation, increasing gradually and reaching the maximum levels at 8-12 h after the experimental manipulation (Table 2; Figure 5).

**Table 1:** The values obtained in the one-way ANOVA for differences associated with time for pups that had access to a lactating doe (NAT) once every 24-h or olfactory stimulated with rabbit maternal milk (M-Milk), with the pheromone 2-methyl-but-2enal (2MB2) or water (H_2_O).

	**NAT**		**M-Milk**		**2MB2**	
	***F(4,28)***	***p<***	***F(4,28)***	***p<***	***F(4,28)***	***p<***
**Liver**	5.1	0.003	1.4	NS	4.4	0.006
**Glucose**	0.7	NS	4.4	0.006	2.6	NS
**Free fatty acids**	0.6	NS	0.8	NS	3.4	0.02
**Triglycerides**	3.6	0.02	0.2	NS	0.3	NS
**Cholecystokinin**	2.4	NS	4.7	0.006	2.6	NS
**Cholesterol**	3.8	0.01	2.1	NS	0.8	NS

The groups were analyzed separately. NS indicates not significant.

**Table 2:** The values obtained in the COSINOR analysis (mesor, acrophase in hours, percent of rhythmicity and probability) for the 24-h rhythmicity of the pups that had access to a lactating doe (NAT) once every 24 h or were olfactory stimulated with rabbit maternal milk (M-Milk), pheromone 2-methyl-but-2enal (2MB2) or water (H_2_O).

	**NAT**			
	***Mesor***	***Acrophase***	***%***	***p<***
**Liver**	4.5	11.6	88.1	0.0008
**Glucose**	17.8	8.1	96.3	0.0002
**Free fatty acids**	1.7	1.8	82.9	0.002
**Triglycerides**	6.2	17.6	90.5	0.0004
**Cholecystokinin**	804.9	3.6	76.9	0.006
**Cholesterol**	8.6	17.6	70.1	0.01
	**M-Milk**			
**Liver**	2.6	8.5	79.7	0.0004
**Glucose**	12.3	9.4	99.7	0.00001
**Free fatty acids**	1.3	7.1	43.1	NS
**Triglycerides**	2.1	2.5	61.2	0.02
**Cholecystokinin**	780.7	-0.7	92.9	0.0001
**Cholesterol**	3.5	10.3	91.3	0.0003
	**2MB2**			
**Liver**	2.7	8.5	96.5	0.0002
**Glucose**	11.7	8	97.6	0.00001
**Free fatty acids**	1.1	1.6	57.2	0.03
**Triglycerides**	2.3	8.4	76.7	0.006
**Cholecystokinin**	713.3	-0.7	82.6	0.002
**Cholesterol**	3.2	11.9	99.7	0.00001
	**H2O**			
**Liver**	2.6	8.8	98.6	0.00001
**Glucose**	11.2	9.1	98.4	0.00002
**Free fatty acids**	1.2	6	49.4	NS
**Triglycerides**	2.5	8.3	94.9	0.00006
**Cholecystokinin**	835.9	-1.4	76.1	0.006
**Cholesterol**	3.2	14.7	89.8	0.0005

The groups were analyzed separately. NS indicates not significant.

Significant rhythmical oscillations in the levels of serum GLU were observed in the M-Milk and H_2_O groups, in contrast the NAT and 2MB2 pups, which only exhibited a tendency of 24-h rhythmicity (Tables 1 and 2; Figure 5). All of the groups exhibited close similarities in the GLU temporal pattern, with the minimum values being observed 2 h before the experimental manipulation, increasing gradually and reaching the maximum levels 8-9 h after the experimental manipulation (Table 2; Figure 5).

Levels of serum FFA exhibited significant rhythmical oscillations only in the 2MB2 group, in contrast the NAT, M-Milk and H_2_O groups, which only showed a tendency of 24-h rhythmicity (Tables 1 and 2; Figure 5). All of the groups exhibited close similarities in the FFA temporal pattern, where the serum levels increased gradually, reaching the maximum 1-7 h after the experimental manipulation, and the minimum levels were observed 14-20 h after experimental manipulation (Table 2; Figure 5).

Levels of serum TAG exhibited significant diurnal fluctuations in the NAT group, in contrast the 2MB2, M-Milk and H_2_O groups, which exhibited a tendency toward 24-h rhythmicity (Tables 1 and 2; Figure 5). In the NAT group, the minimum values of the TAG levels occurred 2 h after doe presentation for nursing, and after that time, the levels gradually increased, reaching the maximum 17 h after nursing (Table 2; Figure 5). In contrast, in the groups of animals that were artificially fed, the temporal pattern of TAG levels were in antiphase, compared to the NAT group. In the M-Milk, 2MB2 and H_2_O groups, the minimum values of the TAG levels occurred 2 h before odor exposure, after which, the levels gradually increased, reaching the maximum 3-8 h after experimental manipulation (Table 2; Figure 5).

For the CCK levels, only the M-Milk group exhibited significant diurnal fluctuations (Tables 1 and 2; Figure 5). In the NAT group, the maximum levels of CCK occurred 3 h after doe presentation for nursing, after which the levels gradually decreased reaching the minimum 16 h after doe presentation for nursing (Table 2; Figure 5). In contrast, in the M-Milk, 2MB2 and H_2_O groups, the maximal levels of CCK occurred approximately 1 h before odor exposure, and the minimum levels occurred 10-12 h after experimental manipulation (Table 2; Figure 5). The mean levels of serum CCK were similar in all of the groups examined (Table 3; Figure 5).

**Table 3:** The values obtained in the two-way ANOVA for differences associated with time and experimental manipulation, in pups that had access to a lactating doe (NAT) once every 24 h or were olfactory stimulated with rabbit maternal milk (M-Milk), the pheromone 2-methyl-but-2enal (2MB2) or water (H_2_O).

	**Group**		**Time**		**G x T**	
	***F(3,109)***	***p<***	***F(4,109)***	***p<***	***F(12,109)***	***p<***
**Liver**	57.7	0.0001	12.5	0.0001	2	0.02
**Glucose**	10.3	0.0001	9.4	0.0001	0.3	NS
**Free fatty acids**	4.6	0.005	2.7	0.03	0.8	NS
**Triglycerides**	61.1	0.0001	0.3	NS	3.3	0.006
**Cholecystokinin**	0.8	NS	6.8	0.0001	1.1	NS
**Cholesterol**	168.9	0.0001	3.7	0.007	3.1	0.001

The groups were analyzed separately. NS indicates not significant.

CHO levels exhibited significant diurnal fluctuations in the NAT and H_2_O groups (Tables 1 and 2; Figure 5). In both groups, the minimum values of the CHO levels occurred 0-2 h after the experimental manipulation; these levels gradually increased, reaching the maximum 14-17 h after the manipulation (Table 2; Figure 5). In contrast, in the groups of animals that were exposed to maternal olfactory cues, that is, the M-Milk and 2MB2 groups, the minimum values of the CHO levels occurred 2 h before the odor exposure, and the levels gradually increased, reaching the maximum 10-11 h after odor exposure (Table 2; Figure 5).

A significant reduction in the mean liver weight, and serum levels of GLU, FFA, TAG and CHO were observed in the pups that were fed artificially (M-Milk, 2MB2 and H_2_O groups) compared to the NAT group, in which the pups were nursed by a lactating doe (Table 3; Figure 5).

## Discussion

This study presents the first evidence that maternal olfactory cues modulate the circadian system in a mammal during early stages of development, particularly in the expression of the 24-h pattern of core body temperature and gross locomotor activity.

Similar to previous reports, rabbit pups nursed every 24-h exhibited diurnal rhythm in core temperature and locomotor activity with a clear anticipatory component [11–13,15,35]. Newborn rabbits that were expose every 24-h to maternal olfactory cues (maternal milk or 2-Methylbut-2-enal) exhibited a characteristic diurnal pattern in both parameters with a clear and stable phase relationship between the time of maternal olfactory cue presentation and the maximal temperature and activity. Importantly, all of the animals of these groups exhibited an anticipatory rise in temperature and activity prior to the time of olfactory stimulation, and the duration and magnitude of the anticipatory component was similar to that observed in the newborn rabbits that were nursed every 24-h. In contrast, the group of rabbit pups that did not receive any maternal olfactory cues, exhibited an atypical temporal pattern in both parameters, occasionally exhibiting bimodal patterns, with a lack of an anticipatory component and poor phase control. These data highlighted the relevance of the maternal olfactory cues as non-photic entraining signals for the circadian system involved in the control of rhythmicity at physiological and behavioral levels in newborn rabbits. However, these olfactory signals lacked an effect in metabolic diurnal rhythmicity.

There is no doubt of the biological relevance that the olfactory system has in mammals during early stages of development, particularly in altricial mammals, such as lagomorphs and rodents. This species born with immature central nervous systems, without a functional visual and auditory system and with limited motor abilities [11,13,26,36]. In fact, it has been suggested that the olfactory system in rabbits is functional from the last days of intra-uterine life [33]. One key component for the mother-young interaction is the 2-methylbut-2-enal, this volatile molecule has functional relevance for rabbit survival. In the current study, 2MB2 and maternal milk presentations triggered the typical suckling-related behavior in 7-day-old Chinchilla rabbits in the same manner as previously described in New Zealand rabbits [28], which intensively respond to the 2MB2 from birth to postnatal day 10 [37–39]. Importantly, these responses were triggered selectively in rabbits by the mammary pheromone and not by any novel odorant [40], [Trejo-Muñoz unpublished data]. It has been demonstrated in newborns that a disruption of olfactory inputs, olfactory bulb extirpation or elimination of functional main olfactory epithelium receptors Cnga2, eliminates the ability of the pups to locate, attach to the mother’s nipples and suck, thereby impeding milk intake. Thus, after a couple of cycles, this disruption can cause starvation and the decease of the pups [27,29,41–44]. Experimental evidence indicates the participation of the MOB for mammary pheromone processing because the presentation of 2MB2 induced changes in 2-deoxyglucose uptake and c-Fos induction in this structure [45,46]. In addition, the destruction of the vomeranasal organ did not impair nipple localization in rabbit pups [29], excluding the participation of the accessory system in the detection of the mammary pheromone.

Evidence in the literature supports the idea that olfactory stimuli, such as pheromones and the blend of odors, act as entraining signals for the circadian system of mammals. In adult rodents, these signals affected the expression of daily patterns of body temperature and wheel-running activity [47,48]. Olfactory cues can also modulate the photic and non-photic entrainment of activity in adult rats [49,50]. Recently, it has been reported that adult rodents exhibit circadian rhythmicity in olfactory sensitivity [51,52], which persists even when the SCN is eliminated. Nevertheless, this rhythmicity is dependent on the canonical clockwork because the fluctuations in olfactory sensitivity are lost in mice deficient for *Bmal1* or both *Per1* and *Per2* genes [51].

In a series of elegant experiments, it has been demonstrated that the main olfactory bulb (MOB) functions as an independent pacemaker in adult rodents [2–4]. The MOB contains cells that exhibit autonomous rhythmicity in their molecular profile and firing rate *in vitro* and also display time-regulated responses to odorants [3,4,51]. In addition, the MOB can modulate circadian rhythmicity, because their removal in rodents and primates altered the period of free-running rhythms in temperature and locomotor activity, delayed the onset of activity phase and affected the re-entrainment rates to light-dark cycles [53–56].

In newborn rabbits, it was found that the clock genes *Per1*, *Bmal1* and *Cry1* are expressed abundantly in the MOB and exhibit a consolidated rhythmicity as early as postnatal day 7 [18]. At this time, the molecular clockwork in the SCN is not fully mature [16], indicating that during pre-visual stages of development, the circadian rhythmicity is under the control of extra-SCN, such as the MOB [18]. In a recent study, we determined that 2MB2 modulated the expression of clock proteins in the MOB and in the SCN of rabbit pups, and this effect was time dependent [Trejo-Muñoz et al., unpublished data]; indicating that olfactory maternal cues act directly on the circadian pacemaker contained in the MOB.

The hypothermia observed in newborn rabbits of the H_2_O group was predominantly associated with the lack of maternal olfactory cues. Experimental evidence suggests that odors are capable to modulate the autonomic nervous system (ANS), in adult rodents the olfactory stimulation with grapefruit and lavender induced changes in the heart rate and body temperature [52,57], and in human infants maternal olfactory stimuli, such as areolar secretions from the Montgomery’s glands, modulate the respiratory pattern and heart rate [58]. Thus, is possible that the absence of maternal olfactory cues during the early stages of development could alter the thermoregulatory ability of newborn rabbits by modifying the activation of the ANS.

It was evident that maternal olfactory cues have significant effects in the temporal pattern of locomotor activity of newborn rabbits, and animals that did not receive the maternal olfactory cues showed atypical 24-h organization. These observations differed from a previous report in New Zealand rabbits that suggested that milk administration was sufficient to synchronize the activity pattern [59]. These differences could also be due to the recording methods. In the current study, we used biotelemetry that automatically recorded the individual pups continuously for 24-h, whereas Morgado et al. 2011 [59] performed activity recordings of all the pups in the litter that underwent a gastrostomy by means of a balance and only recorded the weight of the animals at 120, 90, 60, 30 and 15 min before and 15, 30 and 60 min after feeding time.

In relation to the evaluated parameters associated with energy metabolism, the current data showed close similarities to previous reports by Escobar et al. 2000 [14]. These researchers found that glucose and free fatty acids did not exhibit diurnal fluctuations in 7-day-old rabbits that were nursed once every 24-h. Moreover, these authors only described a diurnal pattern in the serum triacylglycerides, where the levels of this metabolite decreased after the time of nursing, and gradually increased until reach its maximum 12-h later. The pups of the NAT group showed a similar tendency, in which the GLU, FFA, CCK and LW did not exhibit 24-h fluctuations, and only TAG exhibited significant rhythmicity, in which the serum levels decreased after nursing, and gradually increased, reaching its maximum 14-h later.

At the metabolic level, maternal olfactory cues appeared to have no effect because the temporal patterns of serum glucose, free fatty acids, triacylglycerides, cholecystokinin and liver weight exhibited close similarities between the groups examined, which suggested that at the peripheral level, the synchronizing cue potentially involves food (milk). This has been widely demonstrated in adult rodents, where the food functions as a potent entraining signal that modifies the temporal organization of the metabolic, behavioral and other circadian parameters [25,60]. In newborn rabbits, it has been suggested that milk administration demonstrates important effects at the metabolic level [14,59], and it is possible that this cue has an effect on peripheral oscillators. Furthermore, these pups may adjust their performance to milk availability or the influence of other maternal cues may be present during nursing. It is important to highlight that the pheromone 2MB2 is present in maternal milk [28], and this molecule could potentially participate as an external entraining signal for peripheral oscillators. Thus, it would be relevant to determine the effect of milk formula supplemented with the 2MB2.

## Conclusions

During pre-visual stages of development, the circadian system of newborn rabbits is sensitive to daily exposure to the maternal olfactory cues contained in milk, particularly to pheromone 2-methylbut-2-enal. The mammary pheromone functions as non-photic entraining signal primarily for the central oscillators that regulate the expression of temperature and behavior. However this effect is partial, since at peripheral level, these cues lack of effect. It is possible that the nursing event is a complex synchronizing cue, which involves other maternal signals such as tactile, thermal and metabolic, which could act at different levels of the circadian system.
